# The impact of digital technological innovation on high-tech industry exports in China

**DOI:** 10.1371/journal.pone.0330494

**Published:** 2025-08-20

**Authors:** Yusen Zou, Jie Liu

**Affiliations:** School of Economics, Guangdong Ocean University, Zhanjiang, Guangdong, China; Southwestern University of Finance and Economics, CHINA

## Abstract

The high-tech industry exports represent a critical breakthrough for China in securing a leading position within the upper echelons of the global value chain, and digital technological innovation (DTI) serves as the primary driving force for enhancing export competitiveness. We conducted an empirical analysis focusing on 31 provinces in China from 2009 to 2022, utilizing patent data related to the digital economy to examine the impact of DTI on high-tech industry exports. Empirical results demonstrate that the DTI significantly enhances the scale of high-tech industry exports, and this positive effect is primarily observed in eastern regions and non-Belt and Road Initiative provinces. The impact channels of DTI primarily involve an increase in the number of high-tech industry firms and the stimulation of innovative behavior within these sectors. The findings provide empirical support for the pivotal role of DTI in the development of a trade powerhouse.

## 1 Introduction

Foreign trade has played a pivotal role in the national economic development since the initiation of China’s reform and opening-up policy. In 2024, China’s exports accounted for 14.5% of the global market share, which remained the world’s largest merchandise trading nation for eight consecutive years. At the same time, the ongoing escalation of domestic factor costs has gradually eroded the competitive advantage of traditional foreign trade sectors primarily characterized by labor-intensive exports. Concurrently, the transformation of the global trade landscape poses a threat to the sustainable growth of international trade and economic stability [1–5]. As a result, the Chinese government has thus actively promoted the upgrading of the trade structure, primarily by expanding the scale of high-tech industry exports [6,7].

The role of the digital economy-driven by technological innovation-in international trade has become increasingly prominent, with China achieving significant breakthroughs in digital technological innovation (DTI). According to data from the China National Intellectual Property Administration, the global number of digital economy invention patent grants in 2023 reached 888,000, with China accounting for 406,000 patents, which represents over 40%. The Chinese government has recognized that the rapid development of the DTI, is crucial for enhancing trade quality. Accordingly, China has implemented policies to incentivize DTI to promote high-tech industry exports. For instance, the Central Committee of the Communist Party of China and the State Council issued the “Opinions on Digital Trade Reform and Innovation Development” in November 2024, to explicitly emphasize strengthening digital technology empowerment in trade to address the instability of external trade environments.

Furthermore, the impact of the digital economy on international trade has become a focal point within academic research. Current studies predominantly examine how the digital economy influences trade costs, trade quality, trade competitiveness, modes of trade, and the value-added of exporting nations [8–11]. While some research has explored the effects of the digital economy on high-tech industry exports [12], similar to other studies, their measurement of the digital economy primarily reflects digital business models represented by internet platforms, the inclusive development of digital infrastructure, and corporate digital transformation initiatives [5,9,13,14]. These approaches fail to analyze the value and effect of DTI, which is this core force within the digital economy.

Certainly, there are existing studies examining the impact of technological innovation on trade [15], primarily focusing on general patent indicators representing broad technological advancements rather than the specific patent counts closely associated with the digital economy. Kong *et al*. [16] assessed DTI by compiling patents related to five key digital technologies-artificial intelligence, blockchain, cloud computing, big data, and the internet of things-though their analysis concentrated on service exports rather than high-tech industry exports.

These studies offer valuable insights for our research. Unlike previous research, we utilize provincial-level patent data from China to develop a specialized metric for measuring DTI and empirically analyze its influence on high-tech industry exports. Our focus on this issue is driven by two key rationales: First, the rise of the digital economy is fundamentally rooted in technological innovation closely linked to digital advancements, which serve as the core drivers of its rapid development. By fostering enterprise incubation and incentivizing innovative activities, DTI can transform trade models. Second, high-tech industry exports are a critical leverage point for optimizing trade structure and enhancing international trade competitiveness. In summary, our study makes two primary contributions:

Firstly, we employed a development metric for the digital economy driven by the fundamental factor of technological innovation. This approach overcomes the limitations of traditional studies that rely solely on internet penetration rates or digital infrastructure indicators. For developing countries, enhancing high-tech industry exports is increasingly urgent for economic growth, industrial upgrading, and global competitiveness. Thus, we present a novel and policy-relevant scholarly contribution. Our findings provide empirical support for developing nations. The spillover effects of DTI enable emerging economies to transcend conventional trade barriers and facilitate industrial upgrading through technological leaps. This offers broadly applicable insights for developing countries.

Secondly, we conducted an in-depth heterogeneity analysis based on our direct examination of the impact of DTI on high-tech industry exports. This provides policy guidelines for optimizing DTI to better support coordinated regional development. Additionally, we identified two synergistic mechanisms through which DTI influences high-tech industry exports. This represents a meaningful extension of Schumpeterian innovation theory in the digital era and offers methodological guidance for investigating the role of the digital economy in international trade.

The remaining structure of our study is organized as follows: Sect 2 presents theoretical analysis and research hypotheses. Sect 3 details the research design. Sect 4 offers empirical results, including baseline regression analysis, endogenous analysis, heterogeneity analysis, and robustness checks. Sect 5 discusses the mechanisms. Sect 6 concludes with policy implications.

## 2 Theoretical analysis and hypothesis

The role of DTI in enhancing trade amount and supporting trade development is widely acknowledged. Compared to general industries, high-tech sectors are more profoundly influenced by digital innovation in technological advancement, production process reengineering, and market competition dynamics. Firms within high-tech industries leverage the informational advantages provided by digital economy platforms to make strategic production and operational decisions at broader spatial-temporal scales. These firms achieve synergistic effects and more efficient resource allocation through cross-regional resource integration, thereby effectively promoting export performance [17]. The inclusive nature of digital technologies also incentivizes domestic high-tech enterprises to expand their export scope and volume [18,19]. As DTI progresses, advanced data analytics and application capabilities facilitate export quality upgrades among trade entities. Moreover, DTI reduces information and search costs in international trade, thereby expanding the scale of high-tech industry exports [20,21].

It is important to note that DTI in China exhibits pronounced regional disparities. Variations in economic endowments and geographical conditions also exert significant influence on export performance [22,23]. For instance, the eastern region outpaces other areas in numerous aspects and demonstrates a robust capacity for technology absorption and transformation. This results in a more pronounced effect of DTI empowerment, thereby exerting a greater stimulative impact on high-tech industry exports [24].

In addition, policy-level interventions can also influence the effectiveness of DTI. Notably, the Belt and Road Initiative (BRI) has had a particularly profound impact on foreign trade, exerting a highly positive effect on the performance of China’s high-tech industry exports [25,26]. For BRI provinces, this may obscure some of the positive impacts of DTI. We propose the following research hypothesis in light of the above analysis.

**Hypothesis 1.** DTI can significantly enhance the high-tech industry exports.

**Hypothesis 2.** In the eastern region, the impact of DTI is more pronounced.

**Hypothesis 3.** In non-BRI provinces, the impact of DTI is more pronounced.

High-tech industries, as knowledge-intensive and technology-intensive sectors, are characterized by high-quality products and robust competitive advantages. The development of these sectors is significantly influenced by factors such as workforce quality, industrial structure, and government support. Additionally, high-tech industries involve substantial investments and risks, with uncertain returns, which results in high entry barriers and stringent product quality standards. The rapid growth of the digital economy has led to reductions in digital infrastructure costs and information acquisition expenses. This change has facilitated the transformation of innovation models and the enhancement of innovation ecosystems [27,28], thereby substantially lowering entry barriers for high-tech industries. The coupling of DTI and high-tech industries continuously generates new business opportunities, expanding the overall number of firms within the sector. Consequently, this increases the supply of high-tech products and promotes export growth.

Furthermore, digital technologies serve as the core drivers of innovation within high-tech industries. These technologies are characterized by rapid iteration, high synergy, and strong permeability, compelling sectoral firms to proactively incorporate digital technologies into their innovation systems. This integration optimizes resource allocation and collaborative innovation networks, which results in a more pronounced digital-driven innovation effect compared to traditional industries [29–32]. Innovation activities in high-tech industries often face financing constraints [33,34]. However, digital technologies can significantly reduce R&D expenditures [35] and alleviate information asymmetry between innovators and financial institutions, thereby supporting innovation funding [36]. Thus, DTI incentivizes enterprises within high-tech sectors to pursue innovative behaviors, further enhancing the market competitiveness of their products and increasing exports [37].

In light of the above analysis, we propose the following research hypothesis regarding the mechanism.

**Hypothesis 4.** DTI can significantly enhance high-tech industry exports by expanding the number of high-tech firms.

**Hypothesis 5.** DTI can significantly enhance high-tech industry exports by stimulating their innovative behaviors.

## 3 Data and methods

### 3.1 Data

Patent data about DTI was sourced from the China Research Data Service Platform (CNRDS). High-Tech industry exports data was obtained from the China Science and Technology Statistical Yearbook. Exchange rate data was provided by the National Bureau of Statistics of China. Provincial-level controls were collected from provincial statistical yearbooks and the China Stock Market & Accounting Research Database (CSMAR). Our final dataset comprises balanced panel data spanning 2009–2022 for 31 Chinese provinces (excluding Hong Kong, Macau and Taiwan), yielding 434 province-year observations.

### 3.2 Indicator construction

Independent Variable, *DTI*_*n*_. We assess the regional DTI capacity by applying a logarithmic transformation to the patent application rate per million inhabitants across provinces, where the rate is adjusted by adding one to the number of patent applications per capita.

Dependent Variable, *Export*. We utilize the logarithmic values of provincial high-tech product exports to quantify the scale of high-tech industry exports. In the mechanism verification, we select the logarithm of the number of high-tech industry firms (*Number*) and the logarithm of the R&D new product development projects per high-tech firm (R&D) as the dependent variables.

Control Variables, we select the exchange rate of the Chinese yuan against the US dollar (*EXR*), provincial non-high-tech industry exports volume (*Exportother*), average years of education (*HR*), infrastructure development level (*INF*), economic development level (*GDP*), economic structure (GDP_sec), and fiscal expenditure (*FIE*). Variable definitions are provided in Table A1.

### 3.3 Model construction

We estimate the following baseline regression model to examine our core research question:

$${\mathrm{Export}}_{i,t}=\beta_{0}+\beta_{1}{\mathrm{DTI}}_{ni,t}+\beta_{2}X_{i,t}+\eta_{t}+\rho_{i}+\varepsilon_{i,t}$$ (1)

In Eq (1), the dependent variable is the scale of high-tech industry exports, denoted as *Export*. The independent variable is the level of DTI in each province (*DTI*_*n*_), with *i* and *t* representing provinces and years, respectively. *X* represents a series of control variables. $\eta_{t}$ accounts for time fixed effects (TE), while $\rho_{i}$ captures provincial fixed effects (PE). $\beta_{0}$ is the intercept term, and $\varepsilon_{i,t}$ denotes the residual disturbance.

In addition, we construct a mediation effect model to examine the underlying mechanisms through which DTI influences high-tech industry exports.

$${\mathrm{Mechanism}}_{i,t}=\alpha_{0}+\alpha_{1}{\mathrm{DTI}}_{ni,t}+\alpha_{2}X_{i,t}+\eta_{t}+\rho_{i}+\varepsilon_{i,t}$$ (2)

$${\mathrm{Export}}_{i,t}=\lambda_{0}+\lambda_{1}{\mathrm{DTI}}_{ni,t}+\lambda_{2}{\mathrm{Mechanism}}_{i,t}+\lambda_{3}X_{i,t}+\eta_{t}+\rho_{i}+\varepsilon_{i,t}$$ (3)

In Eq (2), the dependent variable is the mediating variable, specifically *Number* and R&D, while the independent variable is *DTI*_*n*_. Eq (3) incorporates the two mediating variables into the regression of *Export*, building upon Eq (1). All other variables and notations are consistent with those in Eq (1). Our primary focus is on several key coefficients in Eqs (1), (2), (3). If both $\beta_{1}$ and $\alpha_{1}$ are significantly positive, and $\lambda_{1}$ < $\beta_{1}$ with $\lambda_{2}$ significantly positive, this indicates the presence of a mediating effect.

### 3.4 Summary statistics

Table 1 reports the descriptive statistics of the variables. The means of *Export* and *DTI*_*n*_ are 14.410 and 5.016, respectively. Notably, the substantial standard deviations indicate significant disparities in *DTI*_*n*_, which likely drives considerable differences in high-tech industry exports.

**Table 1:** Summary statistics.

Variable	N	Mean	S.D	Min	Max
*Export*	434	14.410	2.560	6.518	18.940
*Number*	434	7.086	1.831	0.000	11.150
R&D	434	1.422	0.468	0.087	2.748
*DTI*_*n*_	434	5.016	1.463	0.527	8.633
*EXR*	434	1.881	0.039	1.815	1.931
*Exportother*	434	16.160	1.669	11.610	19.760
*HR*	434	9.107	1.153	4.222	12.680
*INF*	434	0.917	0.579	0.022	2.540
*GDP*	434	18.920	1.025	15.300	20.980
GDP_sec	434	42.990	8.806	15.800	59.000
*FIE*	434	0.275	0.202	0.096	1.379

## 4 Results

### 4.1 Baseline regression

Table 2 reports the baseline regression results for Eq (1). Column (1) of the regression results indicates that no control variables are added, column (2) adds some control variables, column (3) further controls for time fixed effects, and column (4) also adds province fixed effects. The results indicate that the coefficients of *DTI*_*n*_ are significantly positive at the 1% level across all four columns. This suggests that higher regional DTI levels are linked to increased high-tech industry exports. Specifically, the regression in column (4) shows that a 1% increase in *DTI*_*n*_ increases *Export* by 0.342%, supporting **Hypothesis 1**.

**Table 2:** The impact of DTI on high-tech industry exports.

	(1)	(2)	(3)	(4)
	*Export*	*Export*	*Export*	*Export*
*DTI*_*n*_	1.306${\;}^{***}$	0.345${\;}^{***}$	0.449${\;}^{***}$	0.342${\;}^{***}$
	(26.666)	(4.692)	(5.297)	(2.673)
*EXR*		-1.818	36.418${\;}^{**}$	35.213
		(-1.524)	(2.054)	(0.724)
*Exportother*		0.419${\;}^{***}$	0.325${\;}^{***}$	0.446${\;}^{***}$
		(6.715)	(4.905)	(4.257)
*HR*		-0.075	-0.084	0.006
		(-0.949)	(-1.022)	(0.026)
*INF*		1.043${\;}^{***}$	0.964${\;}^{***}$	0.390
		(9.357)	(7.960)	(1.070)
*GDP*		0.724${\;}^{***}$	0.938${\;}^{***}$	0.828${\;}^{**}$
		(6.521)	(7.743)	(2.241)
GDP_sec		0.011	0.004	-0.038${\;}^{**}$
		(1.555)	(0.536)	(-2.568)
*FIE*		-1.312${\;}^{**}$	-0.824	-0.943
		(-2.571)	(-1.569)	(-0.696)
*Cons*	7.855${\;}^{***}$	-4.766	-80.511${\;}^{**}$	-75.654
	(29.348)	(-1.519)	(-2.330)	(-0.759)
TE	No	No	Yes	Yes
PE	No	No	No	Yes
*N*	434	434	434	434
${\text{R}}^{2}$	0.557	0.869	0.972	0.954

Note: ${\;}^{*}p<0.10$, ${\;}^{**}p<0.05$, ${\;}^{***}p<0.01$. T-statistics are in parentheses. The same applies hereinafter.

These findings demonstrate DTI’s role in driving continuous global value chain transformation, where regions with advanced DTI tend to occupy high-end positions within the value chain. Furthermore, this underscores DTI as a key driver in international trade competition, which replaces traditional factors like labor and resources.

### 4.2 Endogeneity

The selection of indicators and the construction of models are critical sources of mutual causation and omitted variable bias, which give rise to endogeneity problems that undermine the robustness of baseline regressions. To address these potential endogeneities, we employ two methodological approaches: the use of instrumental variables (IV) and the inclusion of additional control variables.

#### 4.2.1 IV estimation.

The increase in high-tech industry exports reflects an optimization of regional industrial structure, which may in turn promote DTI. An endogenous relationship exists between these variables. Thus, we select an appropriate IV and use the two-stage least squares (2SLS) method.

We consider government policies, given the significant influence of government policies on DTI. Furthermore, Hangzhou holds a leading position domestically in digital economy development. The closer a province is geographically to Hangzhou, the higher its level of DTI. Accordingly, we extract keyword frequencies related to the digital economy from provincial government work reports. By integrating the geographic distance (between provincial capital cities and Hangzhou) and digital policy keyword counts. We further construct the IV, DTI_iv. The calculation is DTI_iv = (frequency of digital economy policy keywords / total word count of the text) /the geographic distance from the provincial capital to Hangzhou.

Table 3 reports the results of the 2SLS regression. The first-stage regression indicates that the coefficient of DTI_iv is significantly positive at the 10% level, which suggests a positive correlation between the instrumental variable and *DTI*_*n*_. Greater government emphasis on digital economic development and a shorter geograohic distance from Hangzhou are associated with higher DTI. The second-stage regression results demonstrates that the coefficient of *DTI*_*n*_ remains significantly positive at the 1% level after addressing endogeneity. They confirms the robustness and reliability of the baseline regression.

**Table 3:** The impact of DTI on high-tech industry exports: IV estimation.

	(1)	(2)
	*DTI*_*n*_	*Export*
DTI_iv	18.165${\;}^{*}$	
	(1.875)	
*DTI*_*n*_		1.778${\;}^{***}$
		(3.308)
*EXR*	2.730${\;}^{***}$	208.872${\;}^{***}$
	(3.297)	(2.656)
*Exportother*	-0.064	0.633${\;}^{***}$
	(-1.527)	(4.012)
*HR*	0.707${\;}^{***}$	-0.341
	(15.710)	(-0.940)
*INF*	0.282${\;}^{***}$	0.218
	(2.865)	(0.539)
*GDP*	0.543${\;}^{***}$	0.117
	(7.490)	(0.241)
GDP_sec	-0.005	-0.075${\;}^{***}$
	(-1.094)	(-3.292)
*FIE*	3.524${\;}^{***}$	-3.777
	(12.000)	(-1.392)
*Cons*	-19.802${\;}^{***}$	-401.542${\;}^{***}$
	(-9.674)	(-2.615)
TE	No	Yes
PE	No	Yes
*N*	420	420
${\text{R}}^{2}$	0.819	0.934

#### 4.2.2 Considering omitted variables.

Although macro-level control variables were incorporated in Eq (1) and fixed effects for time and province were accounted for, some omitted variables inevitably remain. For instance, local economic development is significantly influenced by local officials [38]. Consequently, we manually collected data on local official turnover to further control for the impact of administrative turnover on high-tech industry exports.

Table 4 reports the results. Even after including various combinations of party secretary and governor turnover as control variables, the coefficient of *DTI*_*n*_ remains significantly positive at the 1% level across all three columns. This indicates that the positive effect of DTI on high-tech industry exports persists after controlling for local official replacements. It confirms the robustness and reliability of the baseline regression.

**Table 4:** The impact of DTI on high-tech industry exports: Considering omitted variables.

	(1)	(2)	(3)
	*Export*	*Export*	*Export*
*DTI*_*n*_	0.339${\;}^{***}$	0.340${\;}^{***}$	0.335${\;}^{***}$
	(2.652)	(2.648)	(2.613)
*EXR*	35.761	30.670	30.324
	(0.736)	(0.627)	(0.622)
*Exportother*	0.445${\;}^{***}$	0.447${\;}^{***}$	0.445${\;}^{***}$
	(4.262)	(4.263)	(4.274)
*HR*	0.005	0.020	0.023
	(0.024)	(0.087)	(0.098)
*INF*	0.394	0.403	0.413
	(1.084)	(1.109)	(1.141)
*GDP*	0.825${\;}^{**}$	0.785${\;}^{**}$	0.769${\;}^{**}$
	(2.237)	(2.094)	(2.045)
GDP_sec	-0.037${\;}^{**}$	-0.037${\;}^{**}$	-0.036${\;}^{**}$
	(-2.530)	(-2.527)	(-2.456)
*FIE*	-0.929	-1.038	-1.042
	(-0.690)	(-0.755)	(-0.763)
*Cons*	-76.637	-66.305	-65.372
	(-0.770)	(-0.662)	(-0.654)
TE	Yes	Yes	Yes
PE	Yes	Yes	Yes
turnover of the party secretary	Yes	No	Yes
turnover of the governor	No	Yes	Yes
*N*	434	434	434
${\text{R}}^{2}$	0.954	0.954	0.955

### 4.3 Heterogeneity

The heterogeneous impacts of DTI are due to the geographical disparities. We conducted a heterogeneity analysis from the perspectives of provincial administrative divisions and the BRI.

#### 4.3.1 Provincial administrative divisions.

China’s DTI exhibits pronounced regional disparities. According to the China National Intellectual Property Administration (CNIPA), core digital economy patents in 2023 demonstrate striking geographical concentration: the Beijing-Tianjin-Hebei (21.7%), Yangtze River Delta (26.8%), and Pearl River Delta (22.6%) regions collectively account for 71.1% of valid invention patents, while all other regions comprise just 28.9%. This substantial divide in capacity suggests potentially heterogeneous effects of DTI on high-tech industry exports across regions.

We empirically examine whether the effects of DTI exhibit systematic regional variation. The sample is segmented into four sub-samples: Eastern, Central, Western, and Northeastern regions. Table 5 reports the results for these distinct samples. Notably, the coefficient of *DTI*_*n*_ is significantly positive at the 1% level only in column (1), while the coefficients in columns (2) to (4) are statistically insignificant. This suggests that only high-tech industry exports in the eastern region are positively influenced by advancements in DTI.

**Table 5:** Heterogeneity analysis: East, Central, West and North-East regions.

	(1)	(2)	(3)	(4)
	East region	Central region	West region	North-East region
	*Export*	*Export*	*Export*	*Export*
*DTI*_*n*_	0.658${\;}^{***}$	-0.244	-0.106	0.174
	(4.046)	(-0.608)	(-0.567)	(0.514)
*EXR*	120.677${\;}^{***}$	-922.118${\;}^{***}$	-179.635${\;}^{**}$	-449.988${\;}^{***}$
	(5.904)	(-4.157)	(-2.240)	(-3.994)
*Exportother*	0.571${\;}^{***}$	-0.080	0.694${\;}^{***}$	-0.492
	(3.140)	(-0.427)	(5.152)	(-1.127)
*HR*	0.136	-0.085	-0.097	-0.548
	(0.829)	(-0.184)	(-0.293)	(-0.958)
*INF*	0.428${\;}^{***}$	1.450	0.065	-6.887${\;}^{**}$
	(3.163)	(1.848)	(0.110)	(-2.388)
*GDP*	0.585${\;}^{***}$	-7.818${\;}^{***}$	0.153	-3.781${\;}^{***}$
	(2.897)	(-3.514)	(0.211)	(-3.053)
GDP_sec	0.053${\;}^{***}$	0.045	-0.023	0.071${\;}^{**}$
	(5.793)	(1.823)	(-0.991)	(2.493)
*FIE*	8.447${\;}^{***}$	-32.278${\;}^{***}$	-3.107${\;}^{**}$	-7.899
	(3.478)	(-4.162)	(-2.087)	(-1.392)
*Cons*	-245.442${\;}^{***}$	1934.584${\;}^{***}$	346.402${\;}^{**}$	957.405${\;}^{***}$
	(-6.094)	(4.149)	(2.095)	(4.074)
TE	Yes	Yes	Yes	Yes
PE	Yes	Yes	Yes	Yes
*N*	140	84	154	42
${\text{R}}^{2}$	0.913	0.928	0.962	0.985

The eastern region exhibits distinct advantages in economic development and leads other regions in DTI. Moreover, this region demonstrates stronger capabilities in technology absorption and transformation, particularly reflected in its superior utilization of data resources and knowledge mining within high-tech industries. These advantages amplify the enabling effects of DTI, thereby generating a more pronounced boost to high-tech industry exports [24]. By contrast, other regions lag behind in digital economic development and face challenges in integrating digital technologies into high-tech industrial production processes. Consequently, the potential export-promoting effects on high-tech industries have yet to materialize in these areas. We validate **Hypothesis 2.**

#### 4.3.2 Belt and road initiative.

In 2013, President Xi Jinping proposed the BRI to boost China’s foreign trade and facilitate structural economic reforms and industrial upgrading. Under the BRI, 18 provincial-level regions were designated as key provinces, where high-tech industries received stronger policy support. This may affect high-tech industry exports, potentially altering the role of DTI.

To test whether DTI’s impact varies across BRI provinces, we divided the sample into BRI provinces and non-BRI provinces and conducted subsample regressions. Table 6 reports the results. The coefficients of *DTI*_*n*_ are significantly positive at the 10% level in both column (1) and column (2). However, the coefficient in column (2) is larger. It indicates that the positive effect of DTI on high-tech industry exports is stronger in non-BRI provinces. The BRI enhances high-tech industry exports through trade facilitation, technology exchange, and policy support, thereby limiting the marginal effect of DTI on export growth in BRI provinces. By contrast, DTI plays a more pronounced role in boosting high-tech industry exports in non-BRI provinces. We validate **Hypothesis 3.**

**Table 6:** Heterogeneity analysis: BRI.

	(1)	(2)
	BRI Provinces	non-BRI Provinces
	*Export*	*Export*
*DTI*_*n*_	0.265	0.423
	(1.678)	(1.902)
*EXR*	-18.612	141.785
	(-0.306)	(1.944)
*Exportother*	0.647${\;}^{***}$	0.171
	(4.857)	(1.150)
*HR*	0.387	-0.248
	(1.347)	(-0.744)
*INF*	0.238	0.140
	(0.470)	(0.267)
*GDP*	-0.028	2.487${\;}^{***}$
	(-0.056)	(4.948)
GDP_sec	-0.042${\;}^{**}$	-0.015
	(-2.255)	(-0.658)
*FIE*	-1.505	0.732
	(-1.076)	(0.309)
*Cons*	37.301	-306.198${\;}^{**}$
	(0.299)	(-2.043)
TE	Yes	Yes
PE	Yes	Yes
*N*	224	210
${\text{R}}^{2}$	0.962	0.953

### 4.4 Robustness

To enhance the robustness of baseline regression results, we also conducted a series of robustness tests.

#### 4.4.1 Replacement of the dependent variable.

The development of new products is critical for maintaining competitive advantage in the context of intense international competition. Thus, we utilize the logarithmic measure of exports of new high-tech products (Export_new) as the dependent variable. Table 7 reports the regression results for this new dependent variable. The coefficients of *DTI*_*n*_ are consistently significant positive at the 1% level across columns (1) to (4). It indicates that DTI substantially promotes the exports of new high-tech products.

**Table 7:** Replacement of dependent variable-(Export_new).

	(1)	(2)	(3)	(4)
	Export_new	Export_new	Export_new	Export_new
*DTI*_*n*_	1.723${\;}^{***}$	1.017${\;}^{***}$	1.115${\;}^{***}$	1.016${\;}^{***}$
	(24.339)	(7.594)	(7.260)	(3.953)
*EXR*		3.174	71.480${\;}^{**}$	42.582
		(1.528)	(2.336)	(0.557)
*Exportother*		0.426${\;}^{***}$	0.338${\;}^{***}$	0.548${\;}^{***}$
		(4.095)	(2.728)	(2.692)
*HR*		-0.326${\;}^{**}$	-0.291${\;}^{**}$	0.343
		(-2.296)	(-2.002)	(0.951)
*INF*		0.932${\;}^{***}$	0.857${\;}^{***}$	2.012${\;}^{***}$
		(5.126)	(4.572)	(3.252)
*GDP*		0.864${\;}^{***}$	1.126${\;}^{***}$	-0.822
		(3.848)	(4.213)	(-0.987)
GDP_sec		0.063${\;}^{***}$	0.062${\;}^{***}$	-0.011
		(4.171)	(4.045)	(-0.362)
*FIE*		-0.698	0.487	-0.107
		(-0.552)	(0.355)	(-0.036)
*Cons*	3.996${\;}^{***}$	-22.159${\;}^{***}$	-157.035${\;}^{**}$	-75.326
	(10.089)	(-3.246)	(-2.572)	(-0.478)
TE	No	No	Yes	Yes
PE	No	No	No	Yes
*N*	422	422	422	422
${\text{R}}^{2}$	0.572	0.801	0.805	0.909

#### 4.4.2 Replacement of independent variables.

First, we employ a new independent variable–*DTIG*, defined as the natural logarithm of one plus the number of digital economy-related patents granted per million people in each province. Patent grants serve as an alternative measure of DTI. Table 8 reports the results for this new variable (*DTIG*). The coefficients of *DTIG* are consistently positive and significant at the 1% level across columns (1) to (4), confirming that DTI-when measured by patent grants-still significantly promotes high-tech industry exports.

**Table 8:** Replacement of independent variables-(*DTIG*).

	(1)	(2)	(3)	(4)
	*Export*	*Export*	*Export*	*Export*
*DTIG*	1.312${\;}^{***}$	0.331${\;}^{***}$	0.488${\;}^{***}$	0.467${\;}^{***}$
	(26.574)	(4.597)	(5.338)	(3.318)
*EXR*		-1.687	53.171${\;}^{***}$	64.357
		(-1.402)	(2.832)	(1.313)
*Exportother*		0.418${\;}^{***}$	0.298${\;}^{***}$	0.441${\;}^{***}$
		(6.739)	(4.450)	(4.311)
*HR*		-0.058	-0.083	0.024
		(-0.750)	(-1.013)	(0.108)
*INF*		1.072${\;}^{***}$	0.966${\;}^{***}$	0.441
		(9.886)	(8.054)	(1.246)
*GDP*		0.721${\;}^{***}$	0.954${\;}^{***}$	0.791${\;}^{**}$
		(6.553)	(8.002)	(2.260)
GDP_sec		0.012	0.003	-0.036${\;}^{**}$
		(1.629)	(0.432)	(-2.466)
*FIE*		-1.291${\;}^{***}$	-0.821	-0.642
		(-2.727)	(-1.649)	(-0.520)
*Cons*	8.489${\;}^{***}$	-4.897	-112.426${\;}^{***}$	-131.693
	(34.698)	(-1.538)	(-3.087)	(-1.319)
TE	No	No	Yes	Yes
PE	No	No	No	Yes
*N*	434	434	434	434
${\text{R}}^{2}$	0.555	0.869	0.873	0.955

Alternatively, we adopt the widely used Digital Financial Inclusion Index from Peking University as an alternative measure of DTI [8,39,40]. While our primary metric captures technological innovation, we conduct robustness checks by replacing our main independent variable with the log-transformed Digital Financial Inclusion Index (*DFI*). As reported in Table 9, the coefficients of *DFI* are consistently positive and statistically significant across columns (1) to (4), which indicates that the level of digital economic development significantly promotes high-tech industry exports.

**Table 9:** Replacement of independent variables-(*DFI*).

	(1)	(2)	(3)	(4)
	*Export*	*Export*	*Export*	*Export*
*DFI*	1.116${\;}^{***}$	0.320${\;}^{**}$	1.851${\;}^{***}$	1.139${\;}^{***}$
	(6.324)	(2.009)	(4.040)	(4.003)
*EXR*		0.061	-99.815${\;}^{***}$	-33.729
		(0.035)	(-3.993)	(-1.818)
*Exportother*		0.407${\;}^{***}$	0.312${\;}^{***}$	0.383${\;}^{***}$
		(5.975)	(3.991)	(3.533)
*HR*		0.198${\;}^{***}$	0.100	-0.057
		(2.739)	(1.384)	(-0.249)
*INF*		1.113${\;}^{***}$	1.044${\;}^{***}$	0.384
		(9.085)	(8.336)	(1.294)
*GDP*		0.991${\;}^{***}$	1.051${\;}^{***}$	-0.156
		(7.521)	(7.971)	(-0.361)
GDP_sec		0.017${\;}^{**}$	0.016${\;}^{**}$	-0.012
		(2.084)	(2.060)	(-0.799)
*FIE*		-0.082	-0.262	-2.503
		(-0.165)	(-0.488)	(-1.470)
*Cons*	8.713${\;}^{***}$	88.511	166.399${\;}^{***}$	72.332${\;}^{**}$
	(9.143)	(1.305)	(3.676)	(2.418)
TE	No	No	Yes	Yes
PE	No	No	No	Yes
*N*	360	360	360	360
${\text{R}}^{2}$	0.090	0.856	0.862	0.966

#### 4.4.3 Excluding municipalities.

Given municipalities’ distinct economic structures, functional roles, and greater central government policy support, we exclude municipalities from the sample. Table 10 reports the results. The coefficients of *DTI*_*n*_ are all significantly positive at the 1% level in columns (1) to (4). This indicates that the positive impact of the DTI is still significant after removing the interference from the sample of municipalities.

**Table 10:** Excluding municipalities.

	(1)	(2)	(3)	(4)
	*Export*	*Export*	*Export*	*Export*
*DTI*_*n*_	1.405${\;}^{***}$	0.309${\;}^{***}$	0.444${\;}^{***}$	0.200${\;}^{***}$
	(26.899)	(3.997)	(4.455)	(3.676)
*EXR*		-1.487	47.978	3.718
		(-1.112)	(1.920)	(0.262)
*Exportother*		0.373${\;}^{***}$	0.284${\;}^{***}$	-2.244${\;}^{***}$
		(5.729)	(3.949)	(-18.751)
*HR*		-0.210	-0.097	-0.144${\;}^{**}$
		(-1.956)	(-0.852)	(-2.229)
*INF*		0.638${\;}^{***}$	0.600${\;}^{***}$	0.327${\;}^{***}$
		(3.822)	(3.517)	(3.542)
*GDP*		1.028${\;}^{***}$	1.217${\;}^{***}$	0.302${\;}^{***}$
		(8.198)	(8.743)	(3.097)
GDP_sec		0.004	-0.008	-0.013${\;}^{**}$
		(0.531)	(-0.818)	(-2.089)
*FIE*		-1.450${\;}^{**}$	-0.544	-1.645${\;}^{***}$
		(-2.528)	(-0.889)	(-4.175)
*Cons*	7.396${\;}^{***}$	-8.412${\;}^{**}$	-106.329${\;}^{**}$	-11.039
	(26.996)	(-2.469)	(-2.177)	(-0.395)
TE	No	No	Yes	Yes
PE	No	No	No	Yes
*N*	378	378	378	378
${\text{R}}^{2}$	0.537	0.865	0.869	0.955

## 5 Mechanism analysis

In our theoretical analysis, we examine the mechanisms through which DTI integrates with high-tech industry. We demonstrate that DTI reduces market entry barriers, thereby facilitating new firms creation. Furthermore, DTI enhances the sector’s creative dynamism through advanced technological capabilities [29,32]. These dual channels collectively drive the expansion of high-tech industry exports, which motivates our subsequent empirical investigation.

Table 11 reports the regression results based on the mediating variable-*Number*. Column (1) displays the baseline regression results from Table 2. Column (2) reports the regression results based on Eq (2). Columns (3) and (4) present the results addressing the endogeneity in the regression of Column (2). Column (3) presents 2SLS results using the same instrumental variables as in Table 3 (second-stage results), while Column (4) controls for potential omitted variables (local official turnover).

**Table 11:** Mechanism Test (Mediator Variable - *Number*)

	(1)	(2)	(3)	(4)	(5)
	*Export*	*Number*	*Number*	*Number*	*Export*
*DTI*_*n*_	0.342${\;}^{***}$	0.128${\;}^{***}$	0.272	0.127${\;}^{***}$	0.182
	(2.673)	(4.015)	(1.738)	(3.956)	(1.680)
*Number*					1.249${\;}^{***}$
					(6.940)
*EXR*	35.213	54.328${\;}^{***}$	70.515${\;}^{***}$	53.971${\;}^{***}$	-32.664
	(0.724)	(4.456)	(3.107)	(4.418)	(-0.834)
*Exportother*	0.446${\;}^{***}$	0.077${\;}^{***}$	0.097${\;}^{***}$	0.077${\;}^{***}$	0.350${\;}^{***}$
	(4.257)	(3.148)	(2.918)	(3.104)	(3.969)
*HR*	0.006	-0.128${\;}^{***}$	-0.169${\;}^{***}$	-0.127${\;}^{***}$	0.166
	(0.026)	(-2.762)	(-2.735)	(-2.709)	(0.939)
*INF*	0.390	0.245${\;}^{***}$	0.224${\;}^{***}$	0.248${\;}^{***}$	0.084
	(1.070)	(2.851)	(2.597)	(2.899)	(0.259)
*GDP*	0.828${\;}^{**}$	0.887${\;}^{***}$	0.809${\;}^{***}$	0.881${\;}^{***}$	-0.280
	(2.241)	(7.773)	(6.345)	(7.725)	(-0.700)
GDP_sec	-0.038${\;}^{**}$	-0.002	-0.006	-0.002	-0.035${\;}^{***}$
	(-2.568)	(-0.497)	(-0.984)	(-0.425)	(-2.724)
*FIE*	-0.943	-0.312	-0.640	-0.319	-0.553
	(-0.696)	(-0.715)	(-1.217)	(-0.728)	(-0.479)
*Cons*	-75.654	-115.261${\;}^{***}$	-145.403${\;}^{***}$	-114.474${\;}^{***}$	68.355
	(-0.759)	(-4.569)	(-3.304)	(-4.531)	(0.843)
TE	Yes	Yes	Yes	Yes	Yes
PE	Yes	Yes	Yes	Yes	Yes
turnover of the party secretary	No	No	No	Yes	No
turnover of the governor	No	No	No	Yes	No
*N*	434	434	420	434	434
${\text{R}}^{2}$	0.954	0.991	0.990	0.991	0.959

The *DTI*_*n*_ coefficients in Columns (2) to (4) are significantly positive, indicating that DTI significantly increases the number of firms in high-tech industry. Column (5) reports the regression results based on Eq (3). The coefficient of *DTI*_*n*_ remains significantly positive, and the coefficient of the mediating variable-(*Number*) is also significantly positive. Most importantly, the coefficient of *DTI*_*n*_ in Column (5) is smaller than that in Column (1) (0.182<0.342), which suggests that *Number* indeed serves as a mediating variable.

The deep integration of the digital and real economies facilitates firm transformation and upgrading. A robust digital-physical ecosystem stimulates the establishment of more high-tech firms. The presence of numerous enterprises not only directly increases the supply of high-tech products for export, but also leverages industrial agglomeration effects to reduce costs, decrease uncertainties in the production process, and enhance adaptability to changes in international markets. Ultimately, this strengthens the export competitiveness of high-tech industries. Consequently, we validate **Hypothesis 4.**

Table 12 reports the regression results based on the mediating variable-(R&D). Column (1) displays the baseline regression results from Table 2. Column (2) reports the regression results based on Eq (2). Columns (3) and (4) present the results addressing the endogeneity in the regression of Column (2). Column (3) presents 2SLS results using the same instrumental variables as in Table 3 (second-stage results), while Column (4) controls for potential omitted variables (local official turnover).

**Table 12:** Mechanism Test (Mediator Variable - *R*&*D*)

	(1)	(2)	(3)	(4)	(5)
	*Export*	R&D	R&D	R&D	*Export*
*DTI*_*n*_	0.342${\;}^{***}$	0.360${\;}^{***}$	0.767${\;}^{***}$	0.361${\;}^{***}$	0.313${\;}^{***}$
	(2.673)	(4.547)	(3.529)	(4.535)	(3.058)
R&D					0.157
					(1.749)
*EXR*	35.213	-28.813	23.400	-27.890	-1.701${\;}^{**}$
	(0.724)	(-1.303)	(0.685)	(-1.241)	(-2.215)
*Exportother*	0.446${\;}^{***}$	0.015	0.058	0.015	0.281${\;}^{***}$
	(4.257)	(0.229)	(0.826)	(0.229)	(3.432)
*HR*	0.006	0.058	-0.032	0.055	-0.047
	(0.026)	(0.404)	(-0.189)	(0.379)	(-0.273)
*INF*	0.390	-0.025	-0.028	-0.028	0.617${\;}^{**}$
	(1.070)	(-0.136)	(-0.150)	(-0.154)	(2.486)
*GDP*	0.828${\;}^{**}$	0.139	-0.058	0.149	0.134
	(2.241)	(0.566)	(-0.233)	(0.590)	(0.490)
GDP_sec	-0.038${\;}^{**}$	0.022${\;}^{***}$	0.011	0.021${\;}^{***}$	0.009
	(-2.568)	(2.693)	(1.110)	(2.632)	(0.852)
*FIE*	-0.943	0.559	-0.182	0.578	-0.197
	(-0.696)	(0.543)	(-0.165)	(0.554)	(-0.209)
Cons	-75.654	56.834	-41.367	54.919	-24.877
	(-0.759)	(1.240)	(-0.615)	(1.178)	(-0.397)
TE	Yes	Yes	Yes	Yes	Yes
PE	Yes	Yes	Yes	Yes	Yes
turnover of the party secretary	No	No	No	Yes	No
turnover of the governor	No	No	No	Yes	No
*N*	434	434	420	434	434
${\text{R}}^{2}$	0.954	0.972	0.968	0.972	0.964

Similarly, in columns (2) to (4), the coefficients for *DTI*_*n*_ remain significantly positive. This indicates that DTI can significantly promote innovative behavior within high-tech industries, thereby increasing the number of new product R&D projects in these sectors. Column (5) presents the regression results based on Eq (3). The coefficient for *DTI*_*n*_ remains significantly positive, and the coefficient for the mediating variable R&D is also significantly positive. Notably, the coefficient for *DTI*_*n*_ in column (5) is smaller than that in column (1) (0.313<0.342), which suggests the presence of a mediating effect through R&D.

These results reveal a symbiotic relationship between DTI and the high-tech industry, in which technological advancement significantly enhances the innovation capacity of the high-tech industry [31]. Innovation constitutes the core competitive feature of high-tech industry [41]. Our findings demonstrate that digital-driven R&D activities strengthen comparative advantage and trade competitiveness through new product development [42], ultimately expanding exports. We validate **Hypothesis 5.**

## 6 Conclusion

We develop a novel measure of DTI based on provincial patent applications in China and examine its impact on high-tech industry exports. Our empirical analysis yields three key findings: (1) DTI significantly enhances high-tech industry exports. (2) This positive effect exhibits regional heterogeneity, being particularly pronounced in eastern regions and non-BRI provinces. (3) We identify two mechanisms: (i) stimulating new high-tech firms and (ii) boosting R&D intensity.

Our conclusions resonate significantly with studies such as Özsoy *et al*. [12] and Jiang and Jia [13], collectively confirming the positive impact of DTI on high-tech industry exports. However, notable differences exist in the theoretical mechanisms and measurement approaches.

Firstly, the differences are reflected in the deepening of theoretical perspectives. Existing literature predominantly explores this topic from the standpoint of digital infrastructure proliferation (e.g., internet penetration rates) or the digitalization of business models (e.g., e-commerce penetration) [5,14]. By contrast, our research focuses on the driving force of the digital economy in technological innovation, specifically represented by patent data, which draws on the core logic of Schumpeterian innovation theory. This shift in perspective explains why traditional metrics, such as the digital finance index, can only capture superficial changes in the business ecosystem [39], whereas patent data provide deeper insights into how technological foundations reshape the export competitiveness of high-tech industries by lowering market barriers **(Hypothesis 4)** and stimulating innovative behaviors **(Hypothesis 5)**.

Secondly, our research subjects are more closely linked to technological innovation. Unlike Kong *et al*. [16], who also focus on DTI but primarily examine the field of service trade exports, our empirical results demonstrate the unique impact of DTI on high-tech industry exports. The export performance of high-tech industries relies heavily on overcoming technological barriers and restructuring production processes-such as in the semiconductor and biopharmaceutical sectors-which are highly synergistic with the deep penetration characteristic of digital technologies [31]. This targeted selection of research subjects enhances both the specificity and depth of our study.

Furthermore, our findings offer new evidence and perspectives for the evolution of Schumpeterian innovation theory in the digital era. Traditionally, this theory emphasizes that innovation reshapes market structures through “creative destruction”. However, our research reveals that technological innovation in the digital era exhibits a dual-pathway dynamic. On one hand, there is the destructive pathway: DTI significantly reduce information costs [20], eroding capital and knowledge barriers in high-tech industries and enabling new entrants to disrupt established monopolies. On the other hand, there is the creative pathway: DTI compels firms to accelerate their own innovation activities, forming a distinctive “DTI → firm innovation → export competitiveness” trajectory. This discovery not only enriches the theoretical implications of Schumpeterian innovation theory in the digital age but also provides new theoretical support and practical guidance for understanding and promoting high-tech industry exports.

Based on these findings, we propose the following implications.

Firstly, high-tech industry exports are a critical component of enhancing China’s international trade competitiveness. Therefore, we argue that the Chinese government should prioritize DTI to amplify its role in strengthening trade competitiveness. Specifically, we recommend implementing incentive policies to support digital innovation, such as preferential tax treatment for digital industries and targeted financial resource allocation. The government should also encourage firms to increase R&D investment in digital technologies and cultivate specialized talent. Additionally, efforts should focus on harnessing digital innovation to optimize trade structures-for instance, by promoting digital trade and facilitating a shift from labor-intensive to technology- and knowledge-intensive industries.

Secondly, the government should address the digital divide in regions lagging in digital innovation. While eastern China benefits from higher levels of digital innovation, leading to greater high-tech industry exports, other regions exhibit no significant positive effects due to technological backwardness. Such regional disparities hinder balanced economic development. Thus, policymakers should design and implement regionally balanced policies to accelerate the diffusion of digital innovation to inland areas, thereby boosting high-tech industries. Moreover, greater emphasis should be placed on leveraging digital innovation to stimulate high-tech sector growth in non-BRI provinces, further enhancing China’s openness through expanded international cooperation.

Thirdly, the government should further streamline the channels through which DTI affects high-tech industry exports. This includes improving the business environment, expanding digital infrastructure, and lowering market entry barriers to increase the number of high-tech firms. Concurrently, policies should facilitate the commercialization of DTI, which enhances the applicability and novelty of high-tech products. These measures would spur new product development, ultimately strengthening the global competitiveness of China’s high-tech industries.

## Appendix

**Table A1:** Variable Definitions.

Variable type	Variable symbol	Variable Definition
Core Dependent variable	*Export*	ln (Exports of high-technology products).
Mechanism variables	*Number*	ln (Number of firms in high-tech industries).
	R&D	ln (1+Number of R&D new product development projects in high-tech industries/number of firms in high-tech industries).
Core Independent variable	*DTI*_*n*_	ln (1 + Number of digital economy-related patents filed per million people in each province).
Control variables	*EXR*	ln (CNY/USD exchange rate).
	*Exportother*	ln (Overall export trade by province - export trade of high-tech products by province).
	*HR*	The average years of education per capita in each province. (Number of primary school-age population×6 + number of junior high school-age population×9 + number of high school-age population×12 + number of college and above-age population×16) / total population aged 6 and above.
	*INF*	The level of infrastructure construction in each province. (Railway operation mileage + highway mileage + inland waterway mileage)/ area of each province.
	*GDP*	Level of economic development. The logarithmic value of gross domestic product.
	GDP_sec	Economic structure. The proportion of the secondary industry in GDP.
	*FIE*	Fiscal expenditure. The proportion of the fiscal expenditure in GDP.

## Supporting information

Data The main data in this article. (XLSX)

## References

[1] Jiang L, Lu Y, Song H, Zhang G. Responses of exporters to trade protectionism: inferences from the US-China trade war. Journal of International Economics. 2023;140:103687. doi: 10.1016/j.jinteco.2022.103687

[2] Fajgelbaum P, Goldberg P, Kennedy P, Khandelwal A, Taglioni D. The US-China Trade War and Global Reallocations. American Economic Review: Insights. 2024;6(2):295–312. doi: 10.1257/aeri.20230094

[3] Chor D, Li B. Illuminating the effects of the US-China tariff war on China’s economy. Journal of International Economics. 2024;150:103926. doi: 10.1016/j.jinteco.2024.103926

[4] Liu Y, Tang T, Ah R, Luo L. Has digital technology promoted the restructuring of global value chains? Evidence from China. Economic Analysis and Policy. 2024;81:269–80. doi: 10.1016/j.eap.2023.11.012

[5] Liang S, Tan Q. Can the digital economy accelerates China’s export technology upgrading? Based on the perspective of export technology complexity. Technological Forecasting and Social Change. 2024;199:123052. doi: 10.1016/j.techfore.2023.123052

[6] Heshmati A, Peng S. International trade and its effects on economic performance in China. China Econ Policy Rev. 2012;01(02):1250009. doi: 10.1142/s1793969012500094

[7] Han M, Zhou Y. The impact of high-tech product export trade on regional carbon performance in China: the mediating roles of industrial structure supererogation, low-carbon technological innovation, and human capital accumulation. Environ Sci Pollut Res Int. 2022;29(21):31148–63. doi: 10.1007/s11356-021-17252-5 35006567 PMC8744375

[8] Li H, Lin X, Zhang Z. How does digital finance affect imports, exports and trade balance: evidence from China. International Review of Economics & Finance. 2025;99:104054. doi: 10.1016/j.iref.2025.104054

[9] Zhang C, Qiu P, Zhang L, Hong X, Wang D. The impact of digital transformation on enterprises’ export stability: evidence from listed companies in China. International Review of Financial Analysis. 2024;96:103582. doi: 10.1016/j.irfa.2024.103582

[10] Ou X, Zhao W, Zheng Z, Mohiuddin M. Towards green trade: digital economy and export quality of green products. Research in International Business and Finance. 2025;75:102777. doi: 10.1016/j.ribaf.2025.102777

[11] Xiaoli M, Benye S, Hongliang L, Raza A. Domestic value chain, digital finance and the quality of firm’s export products. Research in International Business and Finance. 2025;77:102877. doi: 10.1016/j.ribaf.2025.102877

[12] Özsoy S, Ergüzel OŞ, Ersoy AY, Saygılı M. The impact of digitalization on export of high technology products: a panel data approach*. The Journal of International Trade & Economic Development. 2021;31(2):277–98. doi: 10.1080/09638199.2021.1965645

[13] Jiang M, Jia P. Does the level of digitalized service drive the global export of digital service trade? Evidence from global perspective. Telematics and Informatics. 2022;72:101853. doi: 10.1016/j.tele.2022.101853

[14] Zhang Q, Duan Y. Digital empowerment and export quality: the moderate effect of market segmentation. Finance Research Letters. 2024;63:105334. doi: 10.1016/j.frl.2024.105334

[15] Palangkaraya A, Jensen PH, Webster E. The effect of patents on trade. Journal of International Economics. 2017;105:1–9. doi: 10.1016/j.jinteco.2016.12.002

[16] Kong N, Wang B, Zhang Y, Zhou N. How does digital technology affect export in services?. Journal of Asian Economics. 2024;95:101814. doi: 10.1016/j.asieco.2024.101814

[17] Hongliang L, Zeren Z, Haoran M. The impact of digital transformation on enterprise export resilience: Evidence from China. International Review of Economics & Finance. 2024;95:103500. doi: 10.1016/j.iref.2024.103500

[18] Brambilla I, Porto GG. High-income export destinations, quality and wages. Journal of International Economics. 2016;98:21–35. doi: 10.1016/j.jinteco.2015.09.004

[19] Brynjolfsson E, Hui X, Liu M. Does machine translation affect international trade? Evidence from a large digital platform. Management Science. 2019;65(12):5449–60. doi: 10.1287/mnsc.2019.3388

[20] Lendle A, Olarreaga M, Schropp S, Vézina P-L. There goes gravity: eBay and the death of distance. Econ J. 2016;126(591):406–41. doi: 10.1111/ecoj.12286

[21] Zhang H, Liu Q, Wei Y. Digital product imports and export product quality: firm-level evidence from China. China Economic Review. 2023;79:101981. doi: 10.1016/j.chieco.2023.101981

[22] Wang Y, Kuang Y. Evaluation, regional disparities and driving mechanisms of high-quality agricultural development in China. Sustainability. 2023;15(7):6328. doi: 10.3390/su15076328

[23] Venables AJ, Limão N. Geographical disadvantage: a Heckscher–Ohlin–von Thünen model of international specialisation. Journal of International Economics. 2002;58(2):239–63. doi: 10.1016/s0022-1996(01)00168-4

[24] Guimón J, Chaminade C, Maggi C, Salazar-Elena JC. Policies to attract R&D-related FDI in small emerging countries: aligning incentives with local linkages and absorptive capacities in Chile. Journal of International Management. 2018;24(2):165–78. doi: 10.1016/j.intman.2017.09.005

[25] Baniya S, Rocha N, Ruta M. Trade effects of the new silk road: a gravity analysis. Journal of Development Economics. 2020;146:102467. doi: 10.1016/j.jdeveco.2020.102467

[26] Zhu X, Gu Z, He C, Chen W. The impact of the belt and road initiative on Chinese PV firms’ export expansion. Environ Dev Sustain. 2023;26(10):25763–83. doi: 10.1007/s10668-023-03705-z

[27] He J (Jack), Tian X. The dark side of analyst coverage: the case of innovation. Journal of Financial Economics. 2013;109(3):856–78. doi: 10.1016/j.jfineco.2013.04.001

[28] Goldfarb A, Tucker C. Digital economics. Journal of Economic Literature. 2019;57(1):3–43. doi: 10.1257/jel.20171452

[29] Wang Q-J, Wang H-J, Chang C-P. Environmental performance, green finance and green innovation: What’s the long-run relationships among variables?. Energy Economics. 2022;110:106004. doi: 10.1016/j.eneco.2022.106004

[30] Hunjra AI, Azam M, Verhoeven P, Taskin D, Dai J. The impact of geopolitical risk, institutional governance and green finance on attaining net-zero carbon emission. J Environ Manage. 2024;359:120927. doi: 10.1016/j.jenvman.2024.120927 38714030

[31] Chemmanur TJ, Loutskina E, Tian X. Corporate venture capital, value creation, and innovation. Rev Financ Stud. 2014;27(8):2434–73. doi: 10.1093/rfs/hhu033

[32] Ling S, Gao H, Yuan D. Catalytic role of the digital economy in fostering corporate green technology innovation: a mechanism for sustainability transformation in China. Economic Analysis and Policy. 2024;84:278–92. doi: 10.1016/j.eap.2024.09.005

[33] Freel MS. Are small innovators credit rationed?. Small Bus Econ. 2006;28(1):23–35. doi: 10.1007/s11187-005-6058-6

[34] Liu J, Xu K, Li J. Public development finance and firm innovation. China Economic Quarterly International. 2024;4(4):266–80. doi: 10.1016/j.ceqi.2025.02.001

[35] Guo B, Wang Y, Zhang H, Liang C, Feng Y, Hu F. Impact of the digital economy on high-quality urban economic development: evidence from Chinese cities. Economic Modelling. 2023;120:106194. doi: 10.1016/j.econmod.2023.106194

[36] Qiao P, Liu S, Fung H-G, Wang C. Corporate green innovation in a digital economy. International Review of Economics & Finance. 2024;92:870–83. doi: 10.1016/j.iref.2024.02.073

[37] Pang S, Li Z, Wang Y. Digital technology and domestic value-added ratio in export: evidence from China’s pilot zones for integrating informatization and industrialization. Economic Analysis and Policy. 2024;84:424–39. doi: 10.1016/j.eap.2024.08.030

[38] Liu J, Zhang Q, Xu K. The influence of private large shareholders on the distribution of bank loan industry: evidence from China. The North American Journal of Economics and Finance. 2023;68:102003. doi: 10.1016/j.najef.2023.102003

[39] Bian Z, Xia M, Zhang Y. High-quality exporting in the digital economy. Economic Analysis and Policy. 2025;86:1198–213. doi: 10.1016/j.eap.2025.04.029

[40] Du Y, Wang Q, Zhou J. How does digital inclusive finance affect economic resilience: Evidence from 285 cities in China. International Review of Financial Analysis. 2023;88:102709. doi: 10.1016/j.irfa.2023.102709

[41] Gustavsson P, Hansson P, Lundberg L. Technology, resource endowments and international competitiveness. European Economic Review. 1999;43(8):1501–30. doi: 10.1016/s0014-2921(98)00027-0

[42] Cassiman B, Golovko E, Martínez-Ros E. Innovation, exports and productivity. International Journal of Industrial Organization. 2010;28(4):372–6. doi: 10.1016/j.ijindorg.2010.03.005

